# Catheter ablation of atrial fibrillation in patients with heart failure and preserved ejection fraction: A meta‐analysis

**DOI:** 10.1002/clc.23841

**Published:** 2022-05-11

**Authors:** Gaoyang Gu, Jing Wu, Xiaofei Gao, Meijun Liu, Chaolun Jin, Yizhou Xu

**Affiliations:** ^1^ Department of the Fourth School of Clinical Medicine Zhejiang Chinese Medical University Hangzhou Zhejiang China; ^2^ Department of Nursing College Zhejiang Chinese Medical University Hangzhou Zhejiang China; ^3^ Department of Cardiology Affiliated Hangzhou First People's Hospital, Zhejiang University School of Medicine Hangzhou Zhejiang China

**Keywords:** atrial fibrillation, catheter ablation, efficacy, heart failure with preserved ejection fraction, safe

## Abstract

**Background:**

Catheter ablation (CA) is an effective treatment for patients with atrial fibrillation (AF). The potential of CA to benefit AF patients with heart failure and preserved ejection fraction (HFpEF) is uncertain.

**Hypothesis:**

CA may be safe and effective for patients with HFpEF.

**Methods:**

The Medline, PubMed, Embase, and Cochrane Library databases were searched for studies evaluating CA for AF patients with HFpEF.

**Results:**

A total of seven trials with 1696 patients were included. Pooled analyses demonstrated similar procedure and fluoroscopy time regarding the use of CA for patients with HFpEF and without HF (weighted mean difference [WMD]: 0.40; 95% confidence interval (CI): −0.01–0.81, *p* = .05 and [WMD: 0.05; 95% CI: −0.18–0.28, *p* = .68]). Moreover, CA was effective in maintaining sinus rhythm (SR) in patients with HFpEF and noninferior for patients without HF [risk ratio (RR): 0.92; 95% CI: 0.76–1.10, *p* = .34). Additionally, CA tended to significantly maintain SR (RR: 4.73; 95% CI: 1.86–12.03, *p* = .001) and reduce rehospitalization for HF compared with medical therapy (RR: 0.36; 95% CI: 0.19–0.71, *p* = .003). However, no significant differences were found between two groups regarding the mortality rate (*p* = .59).

**Conclusion:**

CA is a potential treatment strategy for patients with HFpEF and demonstrates equivalent efficacy to that of patients without HF. Moreover, the benefits of CA in maintaining SR and reducing rehospitalization of HF patients were significantly better than those of medical therapy. Additional randomized controlled trials are warranted to confirm our results.

## 1 INTRODUCTION

The diagnosis of heart failure with preserved ejection fraction (HFpEF) requires the identification of symptoms or signs of heart failure (HF) and preserved left ventricular ejection fraction (LVEF ≥50%), which accounts for approximately half of HF cases.^1^ HFpEF is alway associated with left ventricular (LV) diastolic dysfunction, which results in increased LV end‐diastolic pressure, followed by increased left atrial (LA) filling pressure and atrial wall pressure, which affects the renin‐angiotensin system, calcium handling, profibrotic and proinflammatory pathways, all of which promote electrical and structural remodeling of the atria and contribute to the development of atrial fibrillation (AF).^2^, ^3^, ^4^, ^5^ Therefore, HFpEF and AF frequently coexist and their comorbidity is associated with poor prognosis.^6^, ^7^

Catheter ablation (CA) is a well‐established therapeutic option for AF.^8^ HF may increase the risk of CA complications. However, CA has been shown to decrease AF burden and reduce mortality and rehospitalization for AF patients with HF with reduced EF (HFrEF) without causing a significant increase in the incidence of adverse events.^9^, ^10^ For patients with HFpEF, Androulakis et al used a single‐arm meta‐analysis to evaluate the use of CA,^11^ in addition, some studies and meta‐analysis compared efficacy of CA in AF patients with HFpEF and HFrEF, and showed similar efficacy and safety between two groups.^12^, ^13^, ^14^ However, these studies were insufficient to determine the superiority of CA in these patients. Studies comparing efficacy between HFpEF and those without HF are necessary to illustrate the universality of CA in AF patients with HFpEF. Moreover, the jury is still out on whether CA is superior to medical therapy alone in these populations. Recently, certain studies attempted to explore the application of CA in AF patients with HFpEF and those without HF, and compared efficacy between CA and medical treatment, but the results were inconsistent.^2^, ^15^, ^16^, ^17^, ^18^, ^19^, ^20^ Therefore, this meta‐analysis was performed to evaluate the clinical benefits of CA for AF patients with HFpEF.

## 2 METHODS

### 2.1 Data sources and search strategy

Relevant studies were sourced from Medline, PubMed, Embase, the Cochrane Library, and Elsevier's ScienceDirect databases. The search strategy employed relevant keywords and medical subject heading (MeSH) terms, including the following: ([Atrial fibrillation] OR [AF]) and ([Radiofrequency] OR [RF] OR [Catheter ablation] OR [ablation]) and ([Heart Failure] OR [HF]) and ([Heart failure with preserved ejection fraction] OR [HFpEF]. The literature search was updated in January 2022.

### 2.2 Inclusion and exclusion criteria

Two reviewers (Gao‐yang Gu and Jing Wu) screened and identified studies that met the following inclusion criteria: (a) Patients with drug‐refractory symptomatic AF (b) patients with HFpEF; (c) patients undergoing treatment using CA for the first time; (d) sample size ≥20; (e) studies that provided reliable information regarding the outcomes. The exclusion criteria were as follows: (a) Patients with HFrEF; (b) An equivocal study design or group allocation and (c) conference abstracts, case reports, case series studies, editorials, review articles, or non‐English language articles.

### 2.3 Quality assessment and data extraction

The study quality was evaluated by 2 investigators (Mei‐jun Liu and Chao‐lun Jin) using the Newcastle‐Ottawa Scale (NOS) for observational studies and the Delphi consensus criteria for randomized controlled studies (RCTs). The NOS system consisted of 8 questions with 9 possible points. A star system was used to assess the data according to the selected populations, comparability of the groups, and exposure/outcome of interest. A previous study with NOS ≥7 was considered to be a study of good quality.^21^ The Preferred Reporting Items for Systematic Reviews and Meta‐analyses Amendment to the Quality of Reporting of Meta‐analyses Statement and recommendations from the Cochrane Collaboration in epidemiology were followed. Data extraction was conducted by mutual agreement and all potential disagreements were resolved by consensus.^22^, ^23^

### 2.4 Outcome definitions

Freedom from AF was defined as absence of any symptomatic or asymptomatic atrial arrhythmia lasting >30 s after completing the blanking period (3 months) after CA or medical treatment. Procedure time was defined as the time from the application of local anesthetics to the withdrawal of all catheters. The fluoroscopy time was defined as the time of fluoroscopy from the start to the end of the procedure. Rehospitalization was defined as rehospitalization after discharge due to HF.

### 2.5 Statistical analysis

Statistical analysis was performed by an independent statistician (Jing Wu). Odds ratios (ORs) or risk ratios (RRs) with 95% confidence intervals (CIs) were used as risk estimates and were pooled by the software. Continuous variables were analyzed using weighted mean differences (WMD). Between study heterogeneity was reflected by *I* ^2^ > 50%. A *p* < .05 was considered to indicate a statistically significant difference. In case of no significant statistical heterogeneity, the fixed effects model was preferentially used as the summary measure. In case of statistical heterogeneity, sensitivity analyses were performed to assess the contribution of each study to the pooled estimate by sequentially excluding the individual trial time period and recalculating the pooled RR estimate for the remaining studies. All statistical analyses were performed using RevMan version 5.3 (Nordic Cochrane Center; The Cochrane Collaboration, 2014).

## 3 RESULTS

### 3.1 Study and data selection

**Figure 1:**
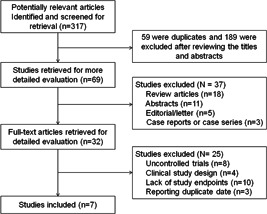
Flow diagram of study selection process

The flowchart of the detailed search process is illustrated in Figure 1. Initially, 317 potential studies were identified, of which 59 were duplicates and 189 were excluded following reviewing of the titles and abstracts. Of the remaining 69 studies, 18 review articles, 11 abstracts, 5 editorial/letters, and 3 case reports were excluded. Furthermore, 25 studies were excluded following a detailed evaluation of the full text for the following reasons: A total of 8 were uncontrolled studies, 4 were clinical study designs, 10 lacked study endpoints, and 3 reported duplicate data. Consequently, 7 clinical trials with 1696 patients were enrolled in the current meta‐analysis.

### 3.2 Study characteristics

The characteristics of the included trials are shown in Table 1. A total of seven trials with 1696 patients were included. Four of these studies compared the clinical outcomes following AF ablation in patients with HFpEF and without HF, whereas the other 3 studies investigated the clinical outcomes of HFpEF patients with AF treated either by CA for AF or medical therapy. The mean age of the study participants ranged from 64 ± 12 years to 74 ± 7 years, and the mean follow‐up duration was from 12 to 50.9 months. The percentage of men was 44%–87.2%. The percentage range of paroxysmal AF was 38%–77%. The majority of the trials included patients that were matched on age, gender, body mass index, and left atrium diameter between the two groups. All studies were rated as having optimal methodological quality. The results of the grouping ensured the feasibility of this meta‐analysis.

**Table 1:** Baseline characteristics of included study

Trial (year)	Treatment group	Patients (*n*)	Age (y)	Male (*n*, %)	BMI (kg/m^2^)	Paroxysmal (*n* %)	CHA2DS2‐VASc	Echocardiographic parameters		Co‐morbidities				Follow (M)	Design	NOS
								LA size, (cm)	LVEF (%)	Hy, (*n* %)	DM, (*n* %)	Pri‐CVA (*n* %)	CAD, (*n* %)			
Cha et al. (2011)^15^	HFpEF + Ablation	157	62.2 (54.4, 70.5)	107 (68)	NR	78 (50)	NR	40 (35, 50)^a^	62 (60, 65)	75 (48)	15 (10)	8 (5)	27 (17)	12	Prospective	9
	No HF + Ablation	100	52.8 (43.9, 59.7)	75 (75)	NR	61 (61)	NR	25 (22, 28)^a^	63 (60, 65)	29 (29)	5 (5)	4 (4)	15 (15)			
Aldaas et al. (2020)^2^	HFpEF + Ablation	51	67.6 (56.6, 74.7)	31 (60.8)	29.7 (24.9, 34.8)	25 (49)	3.0 (2.0, 4.0)	4.2 (3.9, 4.9)	58 (52, 65)	38 (75)	8 (16)	5 (10)	19 (37)	50.9 (24.5, 62.3)	Retrospective	8
	No HF + Ablation	456	64.3 (57.6, 70.5)	307 (67.3)	27.8 (25.0, 31.0)	331 (74)	2.0 (1.0, 3.0)	4.0 (3.7, 4.5)	64 (60, 68)	243 (53)	44 (10)	38 (8)	51 (11)	31.3 (9.2, 57.3)		
Rattka et al. (2020)^18^	HFpEF + Ablation	35	69 ± 9	14 (40)	29 ± 6	27 (77.1)	NR	NR	NR	28 (80.0)	5 (14.3)	2 (5.7)	13 (37.1)	29 ± 20	Retrospective	7
	No HF + Ablation	150	64 ± 12	87 (58)	28 ± 5	105 (70.0)	NR	NR	NR	115 (76.7)	18 (12.0)	14 (9.3)	51 (34.0)			
Yamauchi et al. (2021)^17^	HFpEF + Ablation	293	69.6 ± 7.9	196 (66.9)	24.4 ± 3.9	61 (48.8)	2.80 ± 1.50	43.5 ± 5.7	63.0 ± 6.4	190 (64.8)	64 (21.8)	23 (7.8)	31 (10.6)	12	Retrospective	9
	No HF + Ablation	125	64.2 ± 9.8	109 (87.2)	25.2 ± 3.6	189 (64.7)	1.76 ± 1.43	42.3 ± 6.0	62.8 ± 5.5	66 (52.8)	23 (18.4)	7 (5.6)	11 (8.8)			
Rattka et al. (2021)^16^	HFpEF + Ablation	43	73 ± 7	19 (44)	28 (26, 30)	26 (60)	4 (3, 5)	46.7 ± 7.4	65.3 (55.0,68.0)	39 (91)	10 (23)	7 (16)	28 (65)	35 ± 22	Case‐control	8
	HFpEF + Medical	43	74 ± 7	19 (44)	29 (25, 31)	22 (51)	4 (3, 5)	48.2 ± 7.5	65.6 (58.6,72.2)	39 (91)	11 (26)	2 (5)	27 (63)			
Fukui et al. (2020)^20^	HFpEF + Ablation	35	70 ± 8	23 (66)	NR	14 (40)	NR	42 ± 6	62 ± 8	21 (55)	8 (21)	NA	1 (3)	703 ± 424 d	Retrospective	8
	HFpEF + Medical	50	71 ± 13	32 (64)	NR	19 (38)	NR	43 ± 6	61 ± 9	32 (46)	20 (29)	NA	6 (12)			
Machino‐Ohtsuka et al. (2019)^24^	HFpEF + Ablation	79	68 ± 7	47 (59.5)	24.6 ± 4.2	NA	3.9 ± 1.3	51 ± 21^b^	65 ± 8	59 (74.7)	27 (34.1)	13 (16.5)	10 (12.7)	24 (12, 36)	Retrospective	9
	HFpEF + Medical	79	68 ± 9	49 (62)	24.3 ± 4.3	NA	4.0 ± 1.5	51 ± 21^b^	65 ± 8	62 (78.5)	30 (38.0)	14 (17.7)	10 (12.7)	24 (11, 37)		

*Note*: Values are reported as the mean ± *SD*, medians (interquartile range), or *n* (%).

Abbreviations: BMI, body mass index; CAD, coronary artery disease; d, day; DM, diabetes mellitus; HF, heart failure; HFpEF, heart failure with preserved ejection fraction; Hy, hypertension; LA, left atrium, LVEF, left ventricular ejection fraction; M, month; NOS, Newcastle‐Ottawa Quality Assessment Scale; NR, not recorded; Pri‐CVA, prior cerebral vascular accident; Y, year.

^a^ Evaluate left atrial by LA volume index (cm^3^/m^2^).

^b^ Evaluate left atrial by left atrial volume (cm^2^).

### 3.3 CA therapy for patients with HFpEF versus without HF

**Figure 2:**
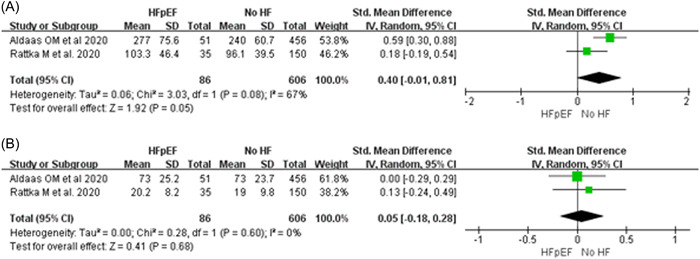
Forest plots of procedural time (A) and fluoroscopy time (B) regarding the use of CA for patients with HFpEF and without HF. CA, catheter ablation; HFpEF, heart failure with preserved ejection fraction

**Figure 3:**
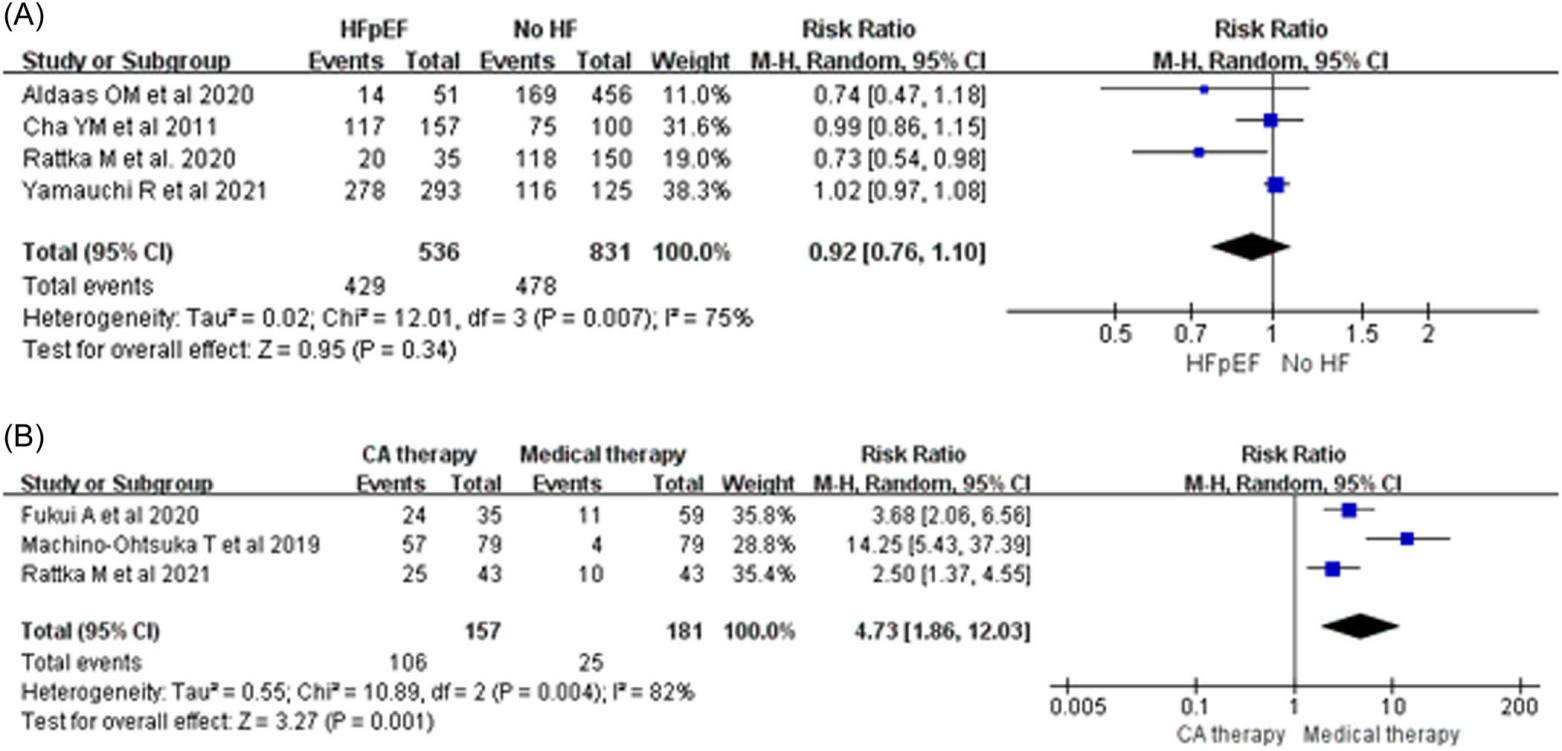
Forest plots of freedom from AF: CA for patients with HFpEF and without HF (A), CA therapy and medical therapy for patients with HFpEF (B). AF, atrial fibrillation; CA, catheter ablation; HFpEF, heart failure with preserved ejection fraction

The pooled analysis demonstrated that HFpEF did not increase the procedure and fluoroscopic time for AF ablation compared with those of patients without HF ([WMD: 0.40; 95% CI: −0.01–0.81, *p* = .05; *I* ^2^ = 67%] and [WMD: 0.05; 95% CI: −0.18–0.28, *p* = .68; *I* ^2^ = 0%]; Figure 2A,B). Moreover, the rate of freedom from AF was also similar between patients with HFpEF and without HF (RR: 0.92; 95% CI: 0.76–1.10, *p* = .34; *I* ^2^ = 75%; Figure 3A).

### 3.4 CA versus medical therapy for patients with HFpEF

**Figure 4:**
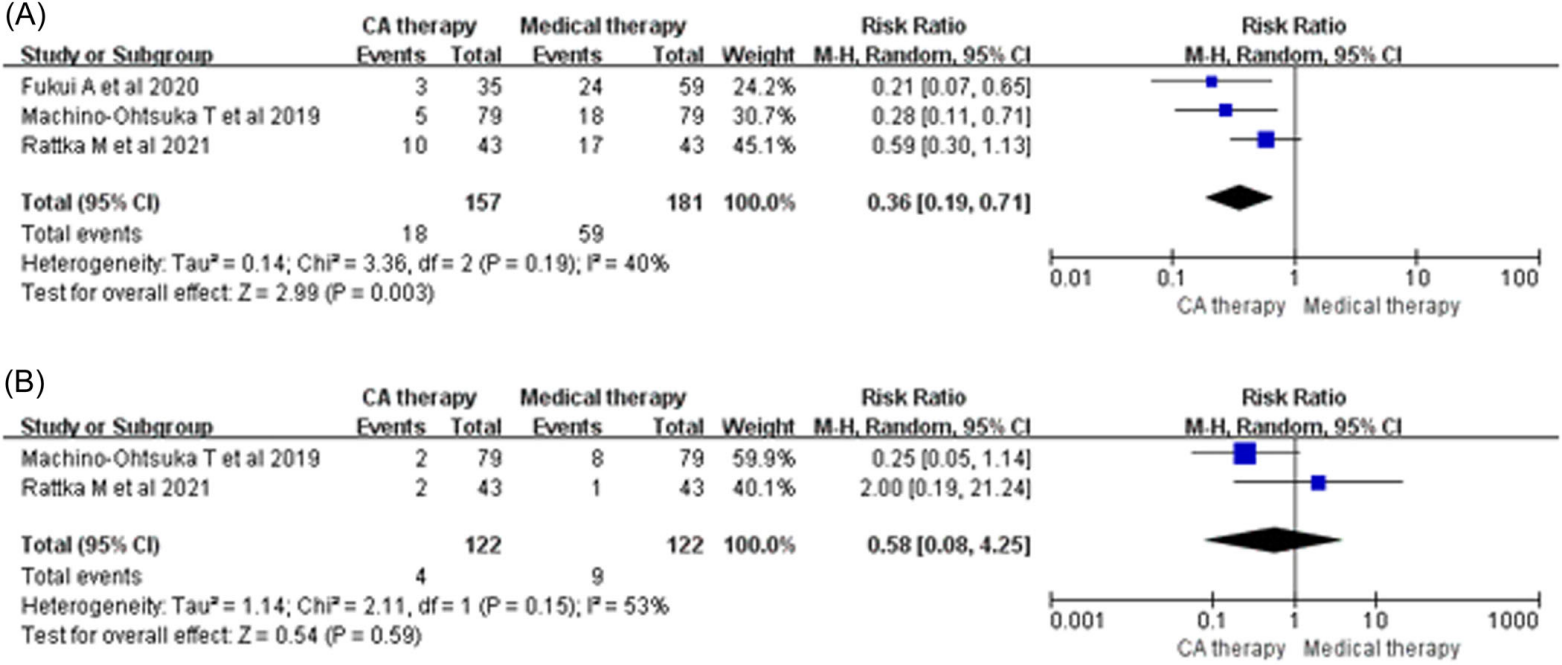
Forest plots of rehospitalization for HF (A) and all‐cause death (B) between CA therapy and medical therapy for patients with HFpEF. CA, catheter ablation; HFpEF, heart failure with preserved ejection fraction

In general, CA can significantly improve the rate of freedom from AF (RR: 4.73; 95% CI: 1.86–12.03, *p* = .001; *I* ^2^ = 82%; Figure 3B) compared with that of medical therapy. In addition, CA therapy can reduce the rate of rehospitalization due to HF (RR: 0.36; 95% CI: 0.19–0.71, *p* = .003; *I* ^2^ = 40%; Figure 4A). However, no significant differences were observed following the comparison of CA with medical therapy with regard to the all‐cause mortality rate (RR: 0.58; 95% CI: 0.08–4.25, *p* = .59; *I* ^2^ = 53%; Figure 4B).

## 4 DISCUSSION

The major findings of the present study are as follows: (a) Patients with HFpEF can benefit from CA compareable with patients without HF with similar procedure and fluoroscopic time and a similar rate of freedom from AF. (b) CA can significantly improve the rate of freedom from AF and the rate of rehospitalization due to HF, while it exhibits a similar rate of all‐cause mortality compared with medical therapy.

HFpEF in patients suffering from AF is associated with increased symptoms and higher morbidity, mortality, and rehospitalization for HF. It has been reported that the prevalence of AF ranges from 30% to 65% for patients with HFpEF.^6^, ^25^ Zakeri et al.^7^ revealed that 2/3 of HFpEF patients would suffer from AF in the disease natural history, which is associated with increased death and HF rehospitalization. Another study by Nagai et al.^26^ demonstrated that AF was a key factor affecting HFpEF development and rehospitalization. Diastolic dysfunction is a hallmark of HFpEF, which leads to intolerance of tachycardia. This accounts for the detrimental effects noted in these patients.^27^ Therefore, certain additional therapeutic strategies that focus on AF are necessary for the treatment of HFpEF patients with AF. The CA of AF in HFrEF patients has been shown to effectively maintain sinus rhythm (SR), improve heart function, and reduce hospitalization and mortality.^9^, ^10^ To explore the efficacy of CA in AF patients with HFpEF, Androulakis et al.^11^ included several studies on the use of CA in AF patients with HFpEF and conducted a single‐arm meta‐analysis. The results showed that CA can be useful to maintain SR. However, as a single‐arm meta‐analysis, it has some limitations, such as the lack of control group and the significant heterogeneity. In addition, some other studies and meta‐analyses compared the efficacy of CA between HFpEF and HFrEF, and identified similar efficacy between two groups.^12^, ^13^, ^14^ These studies tried to explore the clinical benefits of CA in these patients, but were clearly insufficient to demonstrate the universality of CA in AF patients with HFpEF. To further evaluate the role of CA in AF patients with HFpEF, studies comparing efficacy of CA in AF patients with HFpEF and those without HF are warranted, and studies comparing CA with medication are also necessary. Although some studies have carried out related explorations, due to the limitations of the number of studies, sample size, study types (observational studies, retrospective studies) and results inconsistent ect, the level of evidence‐based is still insufficient.^2^, ^15^, ^16^, ^17^, ^18^, ^19^, ^20^ These issues prompted us to conduct this meta‐analysis.

It has been reported that the procedure and fluoroscopic time in patients with HF are significantly longer than those without HF. Substrate remodeling and enlargement of LA may be some of the important reasons.^17^, ^28^ However, our analysis identified similar procedure and fluoroscopic time periods for patients with HFpEF compared with those without HF, which was in agreement with previous studies.^17^, ^18^ The reasons may be as follows: (a) according to the baseline data of included studies, patients with HFpEF did not experience significant enlargement of the LA when compared with patients without HF; (b) patients with HFpEF are less likely to develop irreversible and severe fibrosis in the LA^29^; (c) patients with HFpEF are not accompanied by a significant decrease in LVEF and fewer emergencies that occurred during procedure. The aforementioned factors may contribute to the nonsignificant differences in the procedure and fluoroscopic time observed for patients with HFpEF and without HF. Moreover, CA of AF can be performed safely in HFpEF, with incidence of complications as low as patients without HF.^2^, ^17^, ^18^

Numerous studies have demonstrated that CA can effectively maintain SR for patients without HF compared with medical therap.^30^, ^31^, ^32^ In addition, an accumulated number of studies have recently shown that CA can effectively maintain SR in patients with HF compared with antiarrhythmic drugs.^9^, ^10^, ^33^ Rattka et al.^16^ demonstrated that even with much stricter rhythm monitoring in patients undergoing CA than patients who were on medical therapy, these patients still exhibited significantly lower rates of AF recurrence. Similar results were obtained in our analysis and the data indicated that CA could reduce the recurrence of AF compared with medical therapy alone. In addition, CA could maintain SR to 80% at 1‐year follow‐up in patients with HFpEF who received pulmonary vein isolation, which was similar to the results of the patients without HF.^18^ Pooled analysis of three studies comparing CA in patients with HFpEF and without HF indicated that CA was effective in maintaining SR and noninferior to patients without HF. The main reason may be that the LA in patients with HFpEF did not exhibit severe remodeling, whereas CA for patients with HFpEF even could reverse LA remodeling.^16^

The maintenance of SR is one of the important factors required to reduce HF rehospitalization, regardless of the type of HF.^34^, ^35^, ^36^ CA has been shown to maintain SR effectively, which subsequently leads to a reduction of re‐hospitalizations for HF and reduced mortality in patients with HFrEF.^10^ Machino‐Ohtsuka et al.^24^ reported that CA could maintain SR effectively and that it was associated with a lower risk of HF rehospitalization in patients with HFpEF. Moreover, following a 792 ± 485 day follow‐up, 9% and 48% patients in the CA therapy and medical therapy groups exhibited rehospitalization for HF (*p* < .001).^20^ Pooled analysis of the included studies indicated a significantly lower risk of rehospitalization for HF in the CA therapy group compared with that of the medical therapy group. However, no significant differences were identified between the CA therapy and medical therapy groups with regard to the all‐cause mortality rate.

### 4.1 Limitation

Our study exhibits several limitations. First, publication bias could not be completely excluded and the inclusion of only published data contributed to bias. Second, separate data of paroxysmal (PAF) and persistent AF (PerAF) were not provided in the included studies, so subgroup analyses of PAF and PerAF could not be performed; Third, ablation protocols are not fully consistent across studies, which may bias the results; Fourth, the application of anti‐HF and antiarrhythmic drugs inevitably varies among patients after procedure, which may affect outcomes, Finally, the number of included studies was limited, and most of them were designed as nonrandomized trials. Therefore, more well‐designed and large‐scale RCTs are required to confirm these findings.

## 5 CONCLUSIONS

The current analysis demonstrated that CA is an efficient, safe, and effective therapeutic approach for AF patients with HFpEF. Similar procedure and fluoroscopic time periods were identified regarding the use of CA for patients with HFpEF and without HF. The success rate of maintaining SR in patients with HFpEF was noninferior to those without HF and was significantly better than that of the medical therapy alone group. In addition, CA can reduce rehospitalization due to HF. However, additional multicenter RCTs are required to confirm these results.

## CONFLICTS OF INTEREST

The authors declare no conflicts of interest.

## Data Availability

The data that support the findings of this study are available from the corresponding author upon reasonable request.
